# High-Capacitance Hybrid Supercapacitor Based on Multi-Colored Fluorescent Carbon-Dots

**DOI:** 10.1038/s41598-017-11347-1

**Published:** 2017-09-11

**Authors:** Rukan Genc, Melis Ozge Alas, Ersan Harputlu, Sergej Repp, Nora Kremer, Mike Castellano, Suleyman Gokhan Colak, Kasim Ocakoglu, Emre Erdem

**Affiliations:** 1 Department of Chemical Engineering, Engineering Faculty of Mersin University, Mersin University, TR-33343 Mersin, Turkey; 2 Advanced Technology, Research, and Application Center, Mersin University, TR-33343 Mersin, Turkey; 3 Institut für Physikalische Chemie, Albert-Ludwigs-Universität Freiburg, Albertstr. 21, 79104 Freiburg, Germany; 4 Department of Energy Systems Engineering, Faculty of Technology, Mersin University, TR-33480 Tarsus, Mersin Turkey

## Abstract

Multi-colored, water soluble fluorescent carbon nanodots (C-Dots) with quantum yield changing from 4.6 to 18.3% were synthesized in multi-gram using dated cola beverage through a simple thermal synthesis method and implemented as conductive and ion donating supercapacitor component. Various properties of C-Dots, including size, crystal structure, morphology and surface properties along with their Raman and electron paramagnetic resonance spectra were analyzed and compared by means of their fluorescence and electronic properties. α-Manganese Oxide-Polypyrrole (PPy) nanorods decorated with C-Dots were further conducted as anode materials in a supercapacitor. Reduced graphene oxide was used as cathode along with the dicationic bis-imidazolium based ionic liquid in order to enhance the charge transfer and wetting capacity of electrode surfaces. For this purpose, we used octyl-bis(3-methylimidazolium)diiodide (C8H16BImI) synthesized by N-alkylation reaction as liquid ionic membrane electrolyte. Paramagnetic resonance and impedance spectroscopy have been undertaken in order to understand the origin of the performance of hybrid capacitor in more depth. In particular, we obtained high capacitance value (C = 17.3 μF/cm^2^) which is exceptionally related not only the quality of synthesis but also the choice of electrode and electrolyte materials. Moreover, each component used in the construction of the hybrid supercapacitor is also played a key role to achieve high capacitance value.

## Introduction

Along with the depletion of fossil fuel reserves environmental problems due to evaluated carbon dioxide emission, increase in the population worldwide resulted in an increased energy demand. Development of new technologies providing efficient conversion of sustainable energy resources into usable energy or advanced electric transportation/storage technologies to be used in portable electronics, electric vehicles, and stationary electrical grids has become gradually more important^1–3^. Recent research tendencies are mainly focused on energy conversion and storage devices including lithium batteries, lead-acid batteries, fuel cells and supercapacitors, in which the supercapacitors are featured by their high performance in energy and power density aspects together with the simple operating conditions and long cycling life^4–7^. In a supercapacitor (SC), the surface of electrode usually made from a conductive layer of porous carbon. The specific capacitance of the SC is the order of surface area along with other parameters such as pore size distribution, pore shape and structure, accessibility of the electrolyte, and electrical conductivity^8, 9^. Due to poor electrolyte accessibility to the solid electrode surface and the liquid electrolyte, only a small portion of the theoretical specific capacitance could be achieved. In this regard, recent developments in nanotechnology has opened up new frontiers by using various carbon nanomaterials, mixed-metal oxides, conducting polymers and recently metal-organic frameworks (MOFs) to overcome many of these drawbacks^9–14^. Carbon nanotubes (CNTs) for example offer both high surface area and conductivity^12, 15^. However, the application of CNTs to commercial SC has been limited both by high contact resistance at the electrode–current collector and the high cost and laborsome synthesis routes which constrain the scale-up process for larger scale manufacturing^16, 17^. Graphene nanosheets, on the other hand, gain particular interest due to structured, accessible pores and interlayer spaces possessing higher accessibility of ions^18^. In a study conducted by Zhang *et al*., showed that with a clever functionalization of graphene surface with ZnO, high capacitance values can be achieved together with reversible charging/discharging ability^19^. Reduced graphene oxide (rGO) has electrical conductivity close to that of graphene with the superiority that they could be produced in upscale and a large surface area that can efficiently host the electrolyte ions^20, 21^. Heterostructured electrodes consist of carbon and other nanomaterials with transition metal oxides (e.g., RuO_2_, IrO_2_, MnO_2_, NiO, V_2_O_5_, Co_3_O_4_ and NiCo_2_O_4_) were found to be effective to increase the specific capacitance by appending a high pseudo-capacitance attributed to their multiple valence state changes^9, 12, 22, 23^. Among these investigated metal oxides, manganese dioxide (MnO_2_), a basic transition metal oxide, was widely confirmed as an ideal and alternative capacitive electrode material with lower cost and high environmentally friendly nature^6, 23^. MnO_2_ nanomaterials of various polymorphs can be synthesized and the resulting phases and morphologies are determinative on many properties of the resulting material^24, 25^. Among them, electrodes prepared from one-dimensional (1D) MnO_2_ nanostructures (α-, β-, γ-, and R-MnO_2_) show excellent electrochemical capacitive behavior due to the redox reactions occur on their surface providing a superior electron transport^26^. It was found that electrochemical performance of MnO_2_ depended on the crystal structure and α -MnO_2_ is more suitable to store cations than β-, γ- or R-MnO_2_ due to its larger tunnel size^27^.

Despite their benefits, the limitations in cell capacitance, cycling life and rate performance of α-MnO_2_ electrodes associated with the poor conductivity is still waiting to be improved to meet the requirements of their practical application in SC^28^. Employing carbonaceous materials, conductive polymers and metal oxides to MnO_2_ electrodes were demonstrated to be a reliable strategy to enhance conductivity and specific capacitance^10, 11, 22, 29–32^. Nanocomposites formed by decorating MnO_2_/rGO with silver nanoparticles were reported as they improved the capacitive performance with a specific capacitance reaching to 467.5 F g^−1^ at the scan rate of 5 mV s^−1^ with no significant degeneration after 1000 cycles at the scan rate of 80 mV s^−1^ ^29^. In another study, a high power density electrode constructed by MnO_2_ deposition on the carbon nanotube was reported^15^. Authors claimed a faster electron/ion transfer kinetics and an altered areal capacitance of 1.0 F cm^−2^ at 0.2 A g^−1^ (1.28 mA cm^−2^) that induced by the porous structure of composite formed by a three-dimensional (3D) carbon nanotube cage^15^. Electrodes prepared by conductive wrapping of MnO_2_/graphene nanostructures with carbon nanotubes or conducting polymer have been shown as they greatly improved the conductivity and increased the supercapacitive performances by ∼20% and ∼45%, respectively^32^.

Carbon Dot (C-Dots) are a new class of carbon allotropes formed of densely packed carbon atoms arranged as zero-dimensional (0D) spherical particles with intriguing fluorescent properties^33–35^. The most prominent feature of carbon nanodots as oppose to other carbon nanomaterials is that C-Dots can be synthesized in multi-gram scale simply by heating foods, food wastes, plant extracts, and beverages to high temperatures in water or organic solvents by carbonization of the carbon-rich matrix^36–39^. However, for the synthesis of fluorescent C-Dots with improved fluorescence efficiency, homogeneity in size and in physicochemical properties, it is essential to use a carbon source which is cheap, accessible, easy to handle and non-toxic^40^. Use of passivating agents, mostly polymers, has been extansively used to alter the C-Dot fluorescence^41^. However, this clearly incures an additional cost to the synthesis. C-Dots generally emits fluorescence in the blue range, however, there are examples of C-Dots emitting fluorescence of different colors that can be synthesized by tuning their size and surface properties^42–44^. C-Dots recently have been used in many electronic applications due to their highly tailorable surface chemistry, uniform shape, ultra-small size leading large specific surface area per volume along with their electronic and optical properties. These areas include, but are not limited to the sensors^45^, light emitting diodes^46^ and solar cells^47, 48^. Recently, C-Dots were also considered as new environmentally friendly electrode material that improves the electron transport, ionic motion and enlarges the contact area between electrode and electrolyte in SC^14, 47, 49–52^.

In this work, combination of state-of-art spectroscopic techniques EPR, Raman and impedance spectroscopies were used in order to characterize and detect the paramagnetically active defect centers, vibrational properties of such defects and finally electrical transport properties of defects and capacitance properties of hybrid supercapacitor. Structural defects in the electrode materal playing vital role in the capacitance value of a supercapacitor. Because capacitance is highly dependent not only on the configuration of the oxidation, as it modifies the electrode surface accessibility of the ions but also the art and the concentration of defect centers^13, 53–56^. It has been reported that effects of many parameters, such as carbon hybridization (*sp* ^2^ or *sp* ^3^), pore shape, structural intrinsic defects are extremely important to understand the charge storage mechanism in sub-nanometre pores and to propose strategies to design the next generation of high-capacitance materials and material-electrolyte systems^57^. For this purpose, a novel hybrid supercapacitor constructed using α-MnO_2_ nanorods decorated with C-Dots synthesized from degassed Cola-drink and rGO as electrode material along with a special type of ionic liquid membrane (C8H16BImI) as electrolyte were introduced as a model and its electrical properties were characterized.

## Results and Discussion

### Synthesis and Fluorescent Properties of Carbon Dots

Cola drinks are popular gaseous beverages which are rich in sugar, fructose/glucose syrup, carbon dioxide, caramel, phosphoric acid, caramel, caffeine and some other natural aroma^58^. Thermal synthesis methods used for C-Dot preparation are simple and cost efficient however, they mostly show low synthesis efficiency below 50% with a carbonated bulky waste^59, 60^. More importantly, the fluorescence efficiency of carbon dots are upregulated by use of passivating agents such as polymers, polypeptides and polysaccharides^61^.

**Figure 1:**
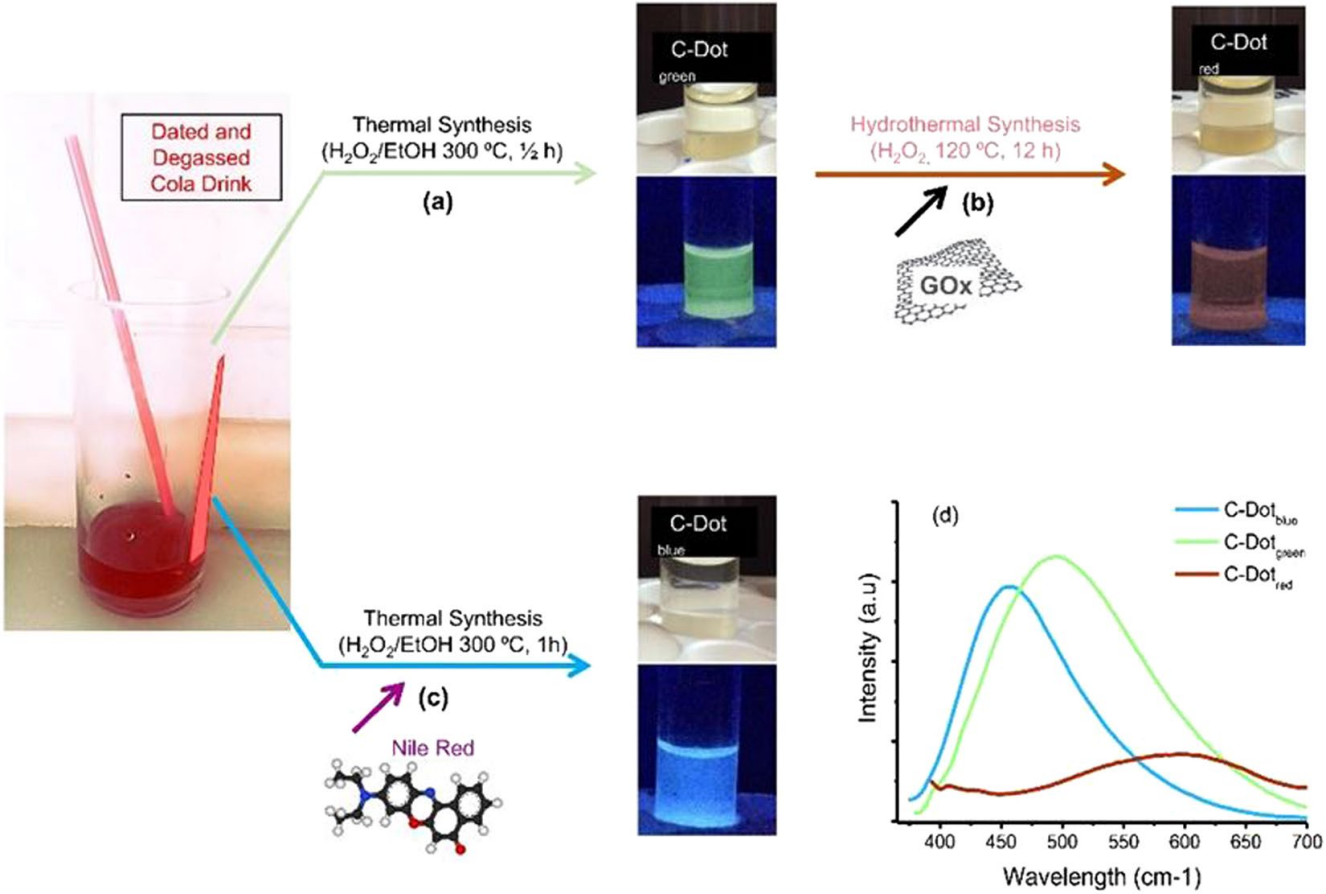
Schematic illustration of the synthesis procedures of (**a**) green, (**b**) red and (**c**) blue emitting fluorescent C-Dots using degassed Cola drink as starting carbon source and representative digital images of as-synthesized C-Dots under bright and UV light (λEx:365 nm, distance = 10 cm). (**d**) Fluorescence emission spectrum of the C-Dots.

Here, we reported the synthesis of water-soluble multi-color fluorescent C-Dots using dated Cola drink as starting material following thermal synthesis method (see the Experimental Section for details) devoiding the use of any additional passivating agent. Instead, here we used the rich ingredient of Cola drink as not only carbon source but also passivating and fluorescence enhancing agent^62^. As explained in Fig. 1a, C-Dots emitting yellowish green fluorescence (C-Dot_green_) were prepared directly by thermal treatment of degassed Cola at 300 °C for 30 min. Particles are collected quite efficiently (67.4%) by centrifugation at 5000 rpm for 10 min^40^. Note that we also have some attempts at different temperatures to produce C-Dots. However, such attempts made at lower temperatures than 300 °C decreased the efficiency of the C-Dots synthesis and at higher temperatures a full carbonization resulted in bulky carbonaceous material rather than C-Dots. To avoid additional parameter to be controlled we kept the heating time constant.

Bright blue fluorescent (Fig. 1b) (*λ*_Ex_:365 nm) but yet water soluble C-Dots were obtained pre-mixing degassed Cola with a hydrophobic and purple colored fluorescent dye (Nile Red). This showed that Nile Red was also contributed to the nanoparticle structure, and both Nile-Red and C-Dot surface energy states were transformed. There are couple of studies on the use of fluorescent dyes (e.g., Fluorescein and Rhodamine B) for tailoring the fluorescence properties of C-Dots by a post-modification of particle surfaces using chemical conjugation steps^63, 64^. Here, we showed that fluorescence character of C-Dots can also be manipulated by direct incorporation of the fluorescence dye during the synthesis avoiding any further surface doping. Red emitting C-Dots were achieved on the other hand by coincidence as a side product of a parallel study on the synthesis of C-Dot embedding GOx composites via hydrothermal treatment (Fig. 1c) of C-Dot_green_ with graphene nanosheets. The washing water collected from the reaction was purified from larger particulates by centrifugation. Resulting carbon dot suspension in pure water showed only a weak red emission under ultra violet (UV) light.

The emission spectra and measured emission maxima (*λ*_max_) of each sample (*λ*_Ex_:365 nm) were plotted in Fig. 1d and values were listed in Table 1. At same C-Dot concentrations (2 mg/mL), C-Dot_green_ possessed the highest photoluminescence (PL) intensity with emission maxima at 495 nm followed by C-Dot_blue_ with a red-shifted emission maximum centered at 435 nm. A poor PL response at shorter wavelengths (475–700 nm) was observed for C-dot_red_ with a red shifted *λ*_max_ in the near-infrared (IR) region (600 nm). This red-shifted fluorescence presumably aroused due to sulfuric acid and potassium permanganate residuals left from graphene oxide^33, 65^.

**Table 1:** Physical properties of synthesized C-Dots.

Sample Code	Carbon Source	*λ*_m_ (nm)	QY (%)	*Rh* (d.nm)	PDI*	Size by TEM (d. nm)	z-Pot (mV)	*σc*(S/cm)
C-Dot_green_	Degassed Cola	498	18.3	12.85 ± 1.3	0.255	≃2–5	−20.8 ± 2.2	150 ± 1.5
C-Dot_red_	C-Dot_green_ + GOx	600	4.60	11.73 ± 1.5	0.158	≃2–7	−48.4 ± 2.1	90 ± 6
C-Dot_blue_	Degassed Cola + Nile Red	435	16.2	42.2 ± 5.6	0.455	≃1–2	−30.1 ± 2.0	130 ± 2.0

Fluorescence emission maxima (λ_m_) and Quantum Yield (QY) measured at *λ*_Ex_:: 365 nm. Hydrodynamic radius (*Rh*) of the C-Dots measured in phosphate buffer saline (PBS, 10x, pH 7.4). Mean diameter of the C-Dots core were calculated by TEM image analysis. Zeta Potential (ζ-Pot) and Electrical conductivity (*σc*) of the samples measured in Milli-Q water. *PDI = Polydispersity Index.

Excitation dependency of the nanodot fluorescence was measured by collecting emission spectra of each C-Dot at changing excitation wavelengths. As shown in Fig. S1 both C-Dot_green_ and C-Dot_blue_ showed excitation dependent fluorescence emission while PL of C-Dot_red_ was found to be independent of the excitation. PL emission of C-Dot_green_ and C-Dot_blue_ can be extended into the near-IR wavelength range by the recombination of localized electron–hole pairs in *sp* ^2^ clusters^66^. Fluorescence quantum yields were then measured by taking quinine sulfate (QS) as a reference and estimated as 18.3%, 16.2% and 4.6% for C-Dot_green_, C-Dot_blue_, and C-Dot_red_, respectively.

### Size and Morphological Characteristics of C-Dots

**Figure 2:**
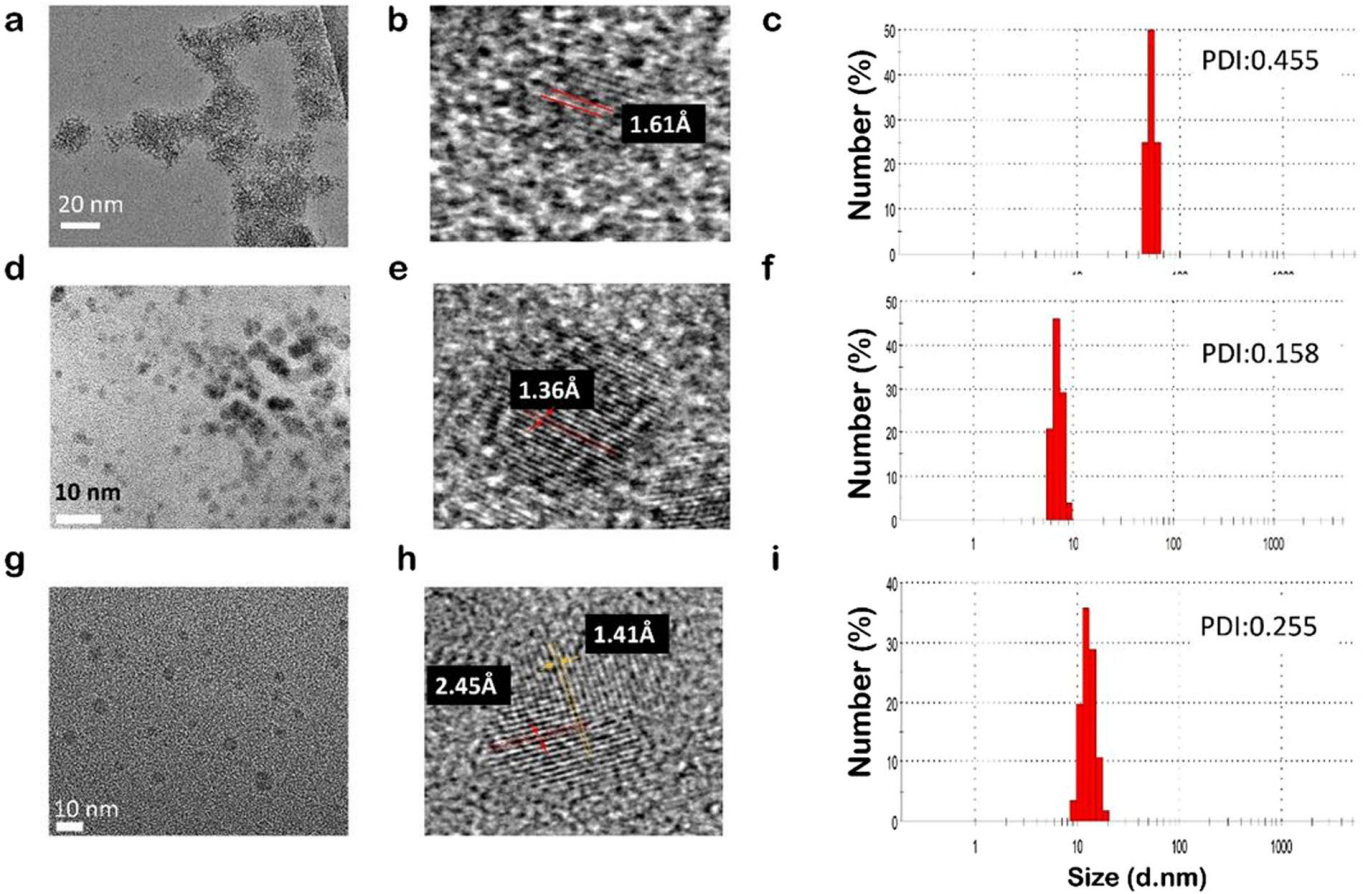
HRTEM images of C-Dots, crystalline lattice of a single nanodot and hydrodynamic size of the C-Dot suspension in water measured using DLS: (**a**,**b** and **c**) for C-Dot_blue_, (**d**,**e** and **f**) for C-Dot_red_ and (**g**,**h** and **i**) for C-Dot_green_.

Table 1 summarizes the several physical characteristics of the resulting C-Dots. Hydrodynamic size and size distribution of C-Dots were evaluated by pursuing dynamic light scattering (DLS) measurements. C-dot_green_ and C-dot_red_ were sized below 15 nm while C-Dot_blue_ has the hydrodynamic size around 45 nm with a polydispersity index (PDI) below 0.5. High-resolution transmission electron microscope (HRTEM) images of resulting C-Dots along with the size distribution graph of each sample were represented in Fig. 2. C-Dot_blue_ were observed as agglomerates with ca. 50 nm diameter, consisting of tiny C-Dots with diameter of ≃1–2 nm (Fig. 1a). C-Dot_green_ and C-Dot_red_ were noted as uniformly distributed individual nanodots with an average size below 5 and 7 nm, respectively. The smaller core size than the size estimated by DLS is due to charge electrical double layer formed by the liquid surrounding the particle.

### Surface Zeta Potential and Electrical Conductivity of C-Dot Suspensions

Surface net charge and electrical conductivity are other important parameters that are essential to predict the colloidal stability, surface and electrical properties of nanomaterials. In the case of carbonaceous materials, these properties may vary between metallic or insulating states depending on the surface defect types and functional groups^67^. As presented in Table 1, all three of the C-Dots exhibited a negative surface charge at pH 4 and C-Dot_red_ has the highest ζ-Pot (−48.4 ± 2.1 mV) due to increased number of acidic functional groups as compared to C-Dot_blue_ (−30.1 ± 2.0 mV) and C-Dot_green_ (−20.8 ± 2.2 mV). Particle conductivity differed in each sample, where C-Dot_green_ showed the highest value (153 S/cm) and the C-Dot_red_ (90 S/cm) have the least. Thus, Dot_green_ is the most suitable candidate material to use as anode material for the hybrid SC compare to red and blue dots while its higher conductivity can be attributed to the high number of intrinsic defects most likely located on the surface which help the electronic transport. Such effect have been recently observed for ZnO nanoparticles^68^ and rGO^69^ as well as ZnO-rGO hybrid^70^. This also explains the higher fluorescence quantum yield of this sample. Although it is difficult to proof, there are some reports indicating that the optical properties have strong influence on the electrical or electrochemical properties of the C-Dots^71, 72^.

### Crystal Structure of C-Dots

The lattice spacing values differ in each C-Dots as shown in Fig. 2b,e and h. C-Dot_green_ contain two different species: 2.45 Å which is the in-plane lattice spacing of graphene ({100} facet)^35^ and 1.41 Å (the C-C bond length in elemental carbon (diamond) that connect the C atoms and are common for the pentagon–hexagon pair^73^. C-Dot_red_ showed a lattice space as short as 1.36 Å which is assigned to the *sp* ^2^ C-C bond length connecting C atoms of two adjacent hexagons. C-Dot_blue_ showed a lattice spacing (1.62 Å) corresponds to a single *sp* ^3^ C-C bond length^74, 75^.

**Figure 3:**
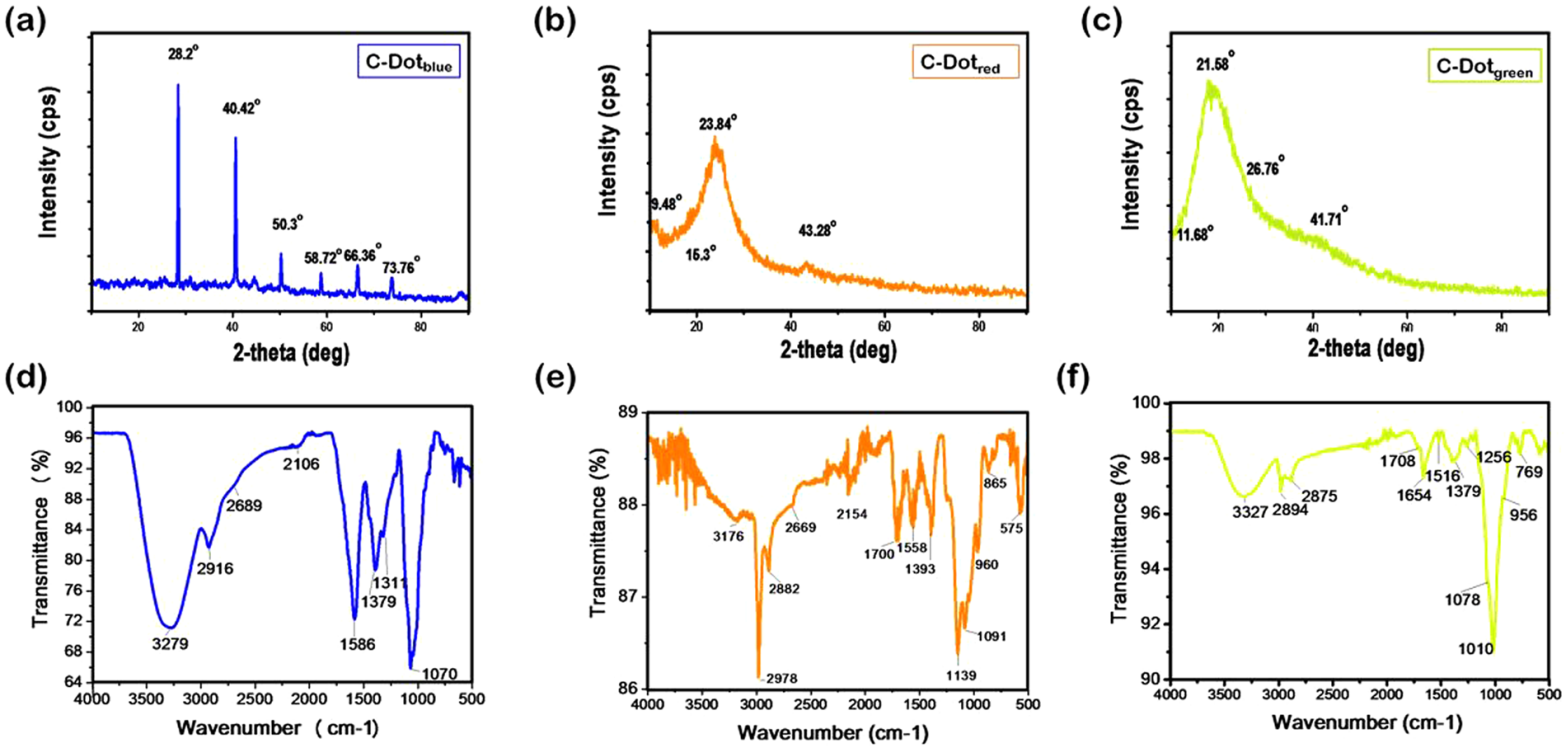
X-Ray diffraction patterns and FT-IR spectrum of C-Dot_blue_ (**a** and **d**), C-Dot_red_ (**b** and **e**) and C-Dot_green_ (**c** and **f**), respectively.

Figure 3a,b, and c represents the X-ray diffraction (XRD) patterns of each CDots. C-Dot_green_ showed a strong peak centered at 19.4° along with a shoulder centered at 41.7°. These peaks are attributed to {100}, {101} planes presenting a higher number of oxygen-containing groups^49, 76, 77^. C-Dot_red_ had three peaks centered at 23.8° with accompanying fairly sharp peak centered at 43.8° of {002} and {101} plane of reduced graphene oxide, respectively. XRD diffractogram show that both C-Dot_green_ and C-Dot_red_ were formed of polyaromatic C domains surrounded with amorphous carbon network^78^. C-Dot_blue_, on the other hand, showed several distinctive peaks correspond to a polycrystalline structure (Fig. 3a)^79^. The peaks centered at 28.2°, 40.2°, 50.3°, 58.7°, and 73.8° were identified as the {002}, {101}, {004}, {103}, and {110} reflections of C-Dots while peak at 66.4° could not be identified. C-Dots are often amorphous and this type of C-Dots with polycrystalline organization is rare in the literature^80^.

### Surface functional groups dominated on C-Dots

Fourier transform-infrared (FT-IR) spectroscopy analysis was performed to determine the acting surface functional groups present on the C-Dots’ surfaces, and corresponding IR spectra were depicted in Fig. 3 and full interpretation was given in the supporting information file. C-Dot_green_ and C-Dot_blue_ displayed a broad peak centered at around 3300 cm^−1^ associated with the hydrogen bonding. The larger hydrodynamic size of the C-Dot_blue_ (Fig. 1c) can be explained by the presence of higher number of water appealing -OH groups on their surface as compared to C-Dot_red_ and C-Dot_green_. FT-IR studies reported here show no contradiction with the literature that shifts in emission maximum often correlated with the increased number of oxidized species on the particle surface which results in a red-shifted PL emission^81^. A comparison of the FT-IR spectra of the three samples reveals that low quantum yield and emission intensity on the other hand correlated with the presence of higher ratio of methyl groups, and domination of C=C bonds as respect to oxygen rich-species^82, 83^. A surface favoring the interfacial hydrogen bonding between C-Dots, decreases the intensity of the fluorescence emitted from those samples due to decreased inter-particle distance between C-Dot pairs (Fig. 1a).

The elemental composition of multi-colored carbon dots, EDX analysis was also performed (Tables S1, 2 and 3). They all show mainly three peaks for ‘C’, ‘O’, ‘P’ and ‘P’ of varying ranges together with some small contamination of other ions.

### Vibrational properties of C-Dots (Raman Analysis)

**Figure 4:**
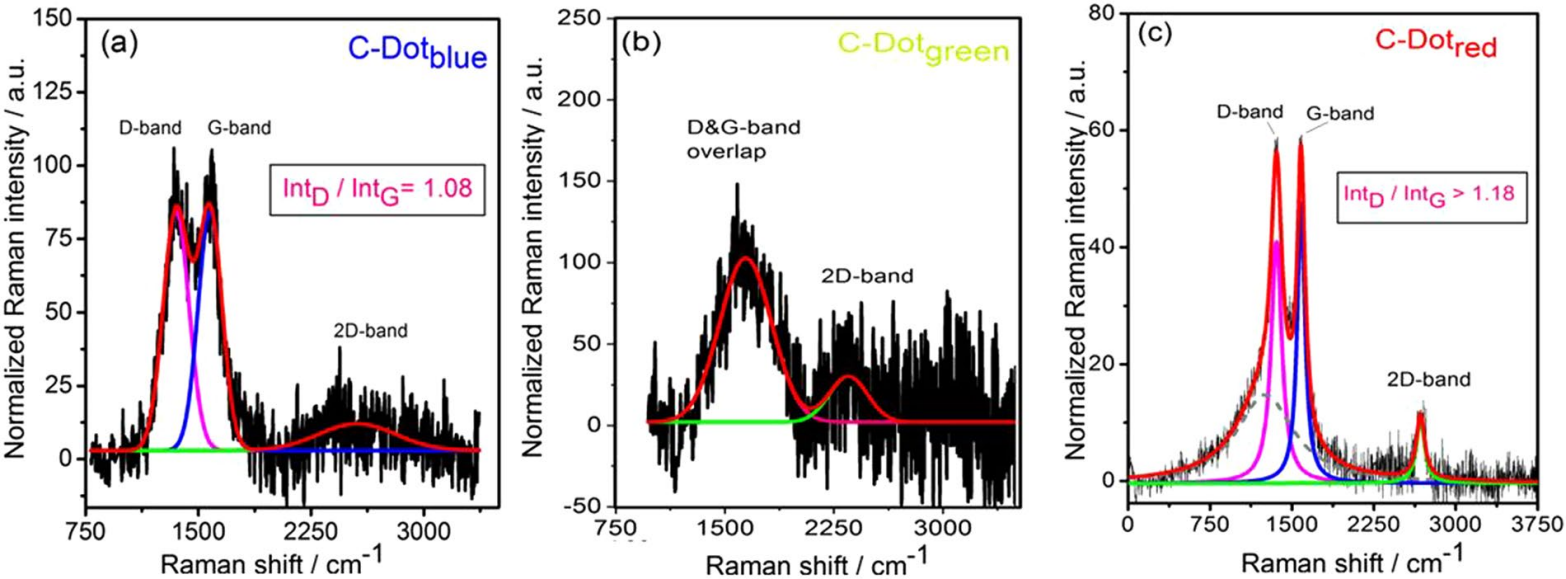
Raman spectra of C-Dot_blue_ (**a**), C-Dot_red_ (**b**) and C-Dot_green_ (**c**), respectively. Intensity ratios for the D- and G- band were calculated by deconvolution of the spectra.

Vibrational properties of C-Dots were analysed by Raman spectroscopy. It is expected that the Raman active carbonaceous materials show two characteristic main peaks, the so-called D and G-band, at around 1350 cm^−1^ and 1600 cm^−1^. The intensity of the D-band is thereby proportional to the amount of disordered sp^3^ carbon; and the intensity of the G-band proportional to the amount of ordered graphitic (*sp* ^2^) carbon contained in the analysed sample. These two correlations represent a possibility to determine the graphitization degree of the synthesized samples by calculating relative intensity ratio of these two bands $(\frac{{{\bf{I}}}_{{\bf{D}}}}{{{\bf{I}}}_{{\bf{G}}}})$. Low values for this ratio would mean that the graphitization degree is elevated, which would theoretically lead to a better electrical conductivity. A third very intense peak at around 3200 cm^−1^ can be observed as well. This dominant, so-called 2D-band is also related to the amount of disordered sp^3^ carbon, but in contrast to the D-band, the intensity of this peak is generally correlated to the performance or intensity of the used laser. Therefore, this 2D-band peak is in general not used for analysing the hybridization behavior or conductivity of the samples. Individual Raman spectra of the samples are shown in Fig. 4a–c. According to this knowledge the blue sample given in Fig. 4a revealed the highest conductivity due to its lowest $(\frac{{{\bf{I}}}_{{\bf{D}}}}{{{\bf{I}}}_{{\bf{G}}}})$ ratio which is 1.08 compare to >1.18 ratio of C-Dot_red_ sample (Fig. 4c). The D- and G-band of C-Dot_green_ sample (Fig. 4b) strongly overlap thus it is not possible to determine the ratio even by deconvolution. This is indication of high disorder or defects. On the other hand, it is also reported that $(\frac{{{\bf{I}}}_{{\bf{D}}}}{{{\bf{I}}}_{{\bf{G}}}})$ ratio is another way of measuring the extent of the disorder and crystallinity. The shift in D and G band of C-Dot_blue_ shows an increased order as compared to C-Dot_red_^66, 84^. Moreover, C-Dots with $(\frac{{{\bf{I}}}_{{\bf{D}}}}{{{\bf{I}}}_{{\bf{G}}}})$ ratios larger than 1 are contributed to the structure defects appeared in the C-Dots^66^. In summary, the Raman spectra presented in Fig. 4 show the existence of defects and also show that how these defect structures affect the vibrational modes of C-Dots. Therefore, we have deeply investigated the defect structures via EPR spectroscopy which is highly sensitive to the detection of paramagnetic defect centers.

### Electron Paramagnetic Resonance (EPR) analysis

It is well known that carbonaceous materials such as graphite, graphene, graphene oxide and as well as C-Dots have intrinsically plenty of EPR active paramagnetic defect centers^69, 70^. These defects mainly arise due to so-called dangling bonds or other defects that possibly generate sub-bandgap defect states. Based on the extraordinary sensitivity and power of EPR spectroscopy on defects, lots of useful microscopic information can be obtained^85^.

**Figure 5:**
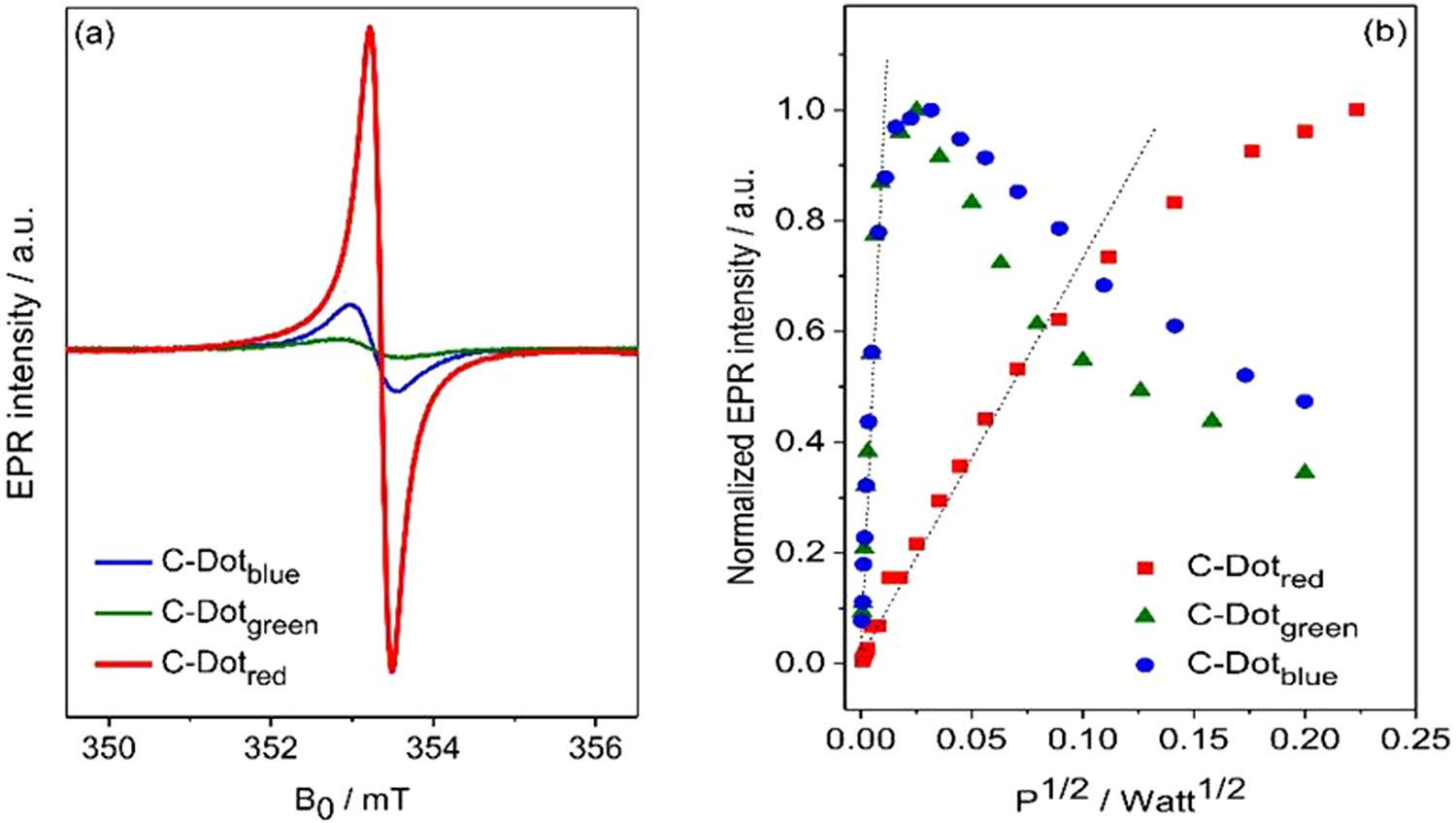
(**a**) Room temperature X-band EPR spectra of C-Dots (blue, green, red). (**b**) The plot of the EPR peak-to-peak intensity with respect to microwave power P^1/2^ for C-Dots.

The observed EPR signals in Fig. 5a can be assigned to either paramagnetic carbon related dangling bond (DB) centers or to other structural intrinsic defects. For the designation of the observed dangling bond centers the *g*-values as determined from EPR data can be used. Carbon radical centers typically occur in an interval of *g*-values ranging from 2.0020–2.0030^86–88^. In addition, by considering the EPR spectral linewidth (ΔB) it may be possible to distinguish between *sp* ^2^- and *sp* ^3^ hybridization. Values for *sp* ^3^-hybridized carbon centers, as found in diamond, for example, have been determined to ΔB(*sp* ^3^) < 1 mT. For graphitic carbon centers with *sp* ^2^-hybridization, generally broader EPR line widths, ΔB(*sp* ^2^) ≥ 1 mT, were reported^86, 89–91^. In the present work, the corresponding variation in g-factors and ΔB is given in Table 2, and according to the abovementioned knowledge we clearly distinguish, experimentally, the difficult issue of defect assignment and carbon hybridization state. The results strongly suggest the existence of the enormous amount of dangling bonds and predominantly occurrence of *sp* ^3^-hybridization. The finding of *sp* ^3^ hybridization is corroborated based on a theory by first principle studies. Nevertheless, it is worth to mention here that the C-Dot_red_ sample nearly approaches to *sp* ^3^ hybridization which also has a strong deviation with respect to *g*-factor as compared to the blue and green C-Dot.

**Table 2:** EPR parameters obtained from the continuous wave X-band EPR spectra presented in Fig. 5(a and b).

	C-Dot_blue_	C-Dot_green_	C-Dot_red_
*g*-factor	2.0029	2.0029	2.0021
Linewidth ΔB (mT)	0.29	0.55	0.93
MW-Power saturation (mW)	0.12	0.12	7.2

Moreover, in order to understand the dependency of carbon related dangling bond defect centers to the microwave power (MW), we have investigated the saturation behavior of EPR signal of all three samples via X-band EPR (Fig. 5b). The peak-to-peak intensities of EPR spectra for all samples rise with increasing microwave power *P*. However, the C-Dot_red_ signal has highly different saturation behavior than other two and deviates strongly at 7.2 mW showing a hard saturation behavior whereas blue and green sample can easily saturate at 0.12 mW (Table 2). Low saturation microwave power is indication of high number of surface defects^85^. This observation represents that we should safely make EPR measurements below saturation values without saturating the signal intensity. Accurate determination of this saturation limit enables us to get off course reliable results about the EPR intensity. Here most importantly to be mentioned is the broader linewidth gives harder saturation points which may be also related to approaching of *sp* ^2^ hybridization in EPR spectral point of view. That shows the unpaired electrons of the defect centers has a totally different electronic environment in C-Dot_red_ as compared to C-Dot_blue_ and C-Dot_green_ ones. This also strongly point out that the reaction to microwave power can be attributed to the localization of defect centers in the C-Dot_red_ samples which are more delocalized electrons (bound states at the defect site) and defect centers in the C-Dot_blue_ and C-Dot_green_ samples are rather localized and located on the surface^85, 92^. Finally, due to its higher conductivity determined during zeta potential measurements, higher disorderness as a result of overlapping of D- and G-band from Raman measurements and its higher defect concentration on the surface from EPR measurements, we conclude that the C-Dot_green_ is the most suitable candidate as a constituent of the anode material to be used in hybrid SC.

### Preparation of C-Dots/MnO_2_ hybrid nanorods and structural characterizations

**Figure 6:**
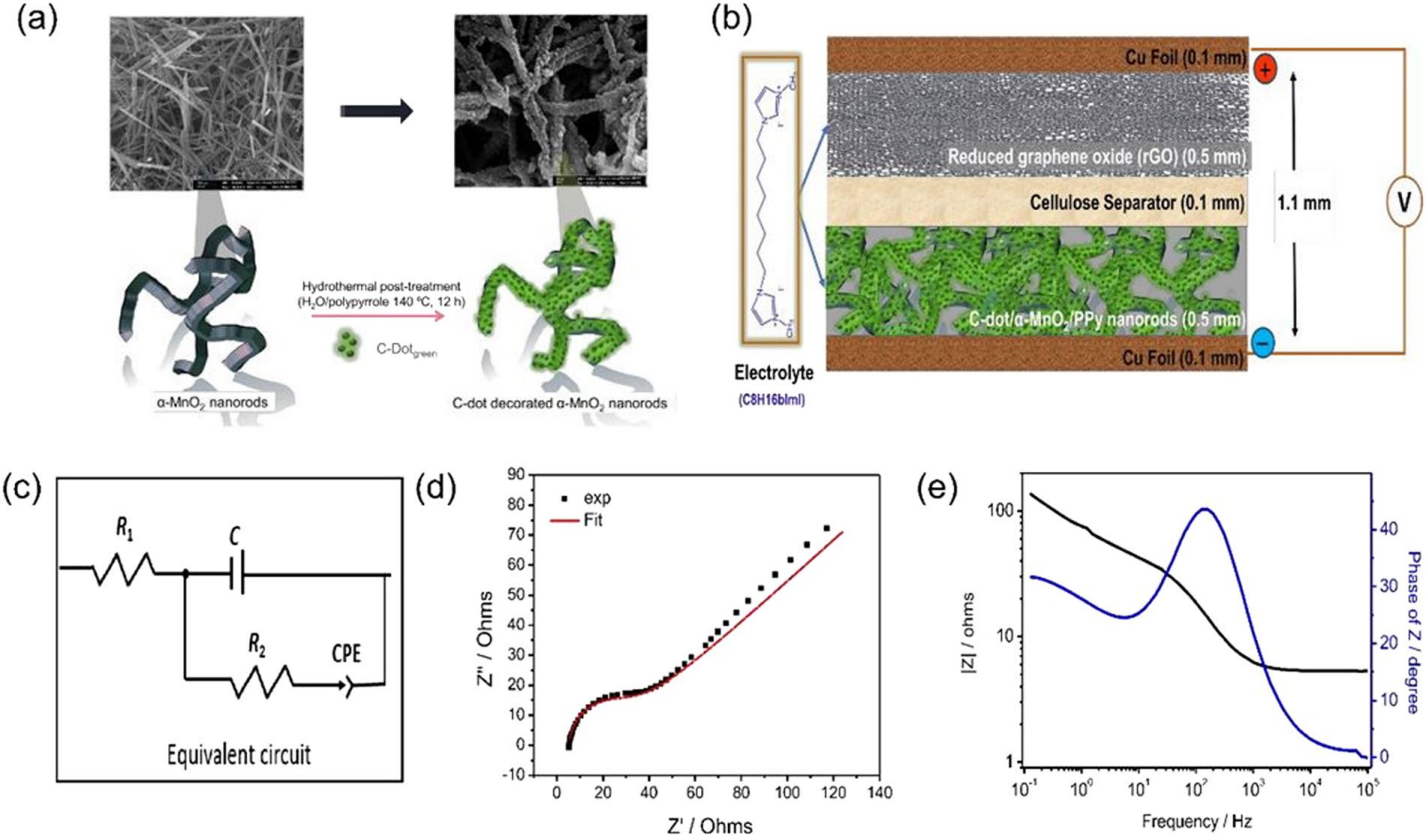
(**a**) Schematic illustrations of the synthesis method of α-MnO_2_/PPy/C-Dot_green_ hybrids from pre-synthesized α-MnO_2_ nanorods and representing scanning electron microscope images. (**b**) Graphical illustration of hybrid supercapacitor model. (**c**) Equivalent modified Randles circuit obtained via fitting of Nyquist plot. (**d**) EIS in Nyquist plot of hybrid supercapacitor. (**e**) The variation of characteristic impedance (absolute value | Z |) with frequency (Bode-plot) for hybrid supercapacitor.

Single-crystal α-MnO_2_ nanorods were prepared by hydrothermal treatment of KMnO_4_ under acidic conditions as described in the experimental part. Figure 6a shows a typical high-magnification SEM image of the MnO_2_ nanorods. They exhibit a uniform size distribution with a width of about 50–150 nm and a length in the range of several micrometers^24, 93^. The XRD analysis indicated that α-MnO_2_ nanorods are highly pure (Fig. S2) and they show a body-centered tetragonal α-MnO_2_ phase (space group I4/m(87)) with lattice constants a) 9.7847 Å and c) 2.8630 Å (JCPDS 44–0141). C-Dot_green_ was further complexed with hydrothermally synthesized α-MnO_2_ nanorods (1:1 w/w) by post-treatment of them hydrothermally in the presence and absence of polypyrrole. Although there is room for the investigation of different mass ratio for maximum supercapacitance performance, in present work we present our lucky-choice ratio which has so far the best results. Presence of polypyyrole could add additional help to enhancement of surface conductivity^94^. S3). Figures 6a and S3c, shows the representative SEM images of α-MnO_2_ samples and their composite with carbon nanodots at different magnifications. α-MnO_2_ nanorods were further decorated with While α-MnO_2_/C-Dot_green_ hybrid nanostructures show similar properties to as-synthesized α-MnO_2_ (Fig. 6a) nanorods, the spherical formations observed on the nanorods in the presence of PPy hybrid structures are noteworthy (Fig. S3). The polymerization of pyrrole on the surface of α-MnO_2_ nanorods enhanced the surface coverage of C-Dots and as a result particle thickness of the rods increased, as clearly be seen in the SEM images shown in Fig. S3d,e ^31, 95^.

### Electrochemical Impedance Spectroscopy (EIS) analysis

Advanced characterization techniques such as Raman, EPR, and impedance spectroscopy can reveal detailed information on the electronic, vibrational, and electrical transport properties. According to Raman (overlapping of D- and G-band) and EPR spectroscopy (higher linewidth compare to blue) results given above C-Dot_green_ includes plenty of functional defects causes the most disorderness. Such defects are crucially important for the charge transport phenomena and the electrical properties such as resistivity (or conductivity). We recently reported for semiconductor ZnO nanocrystals that the defect-charge density has the key role for non-ohmic behavior of due to the defect distribution and interactions in the surface structure^68^. In order to demonstrate such effect, we measured the electrical conductivity of α-MnO_2_/PPy nanorods before/after doped with C-Dots. The electrical conductivity of nanorods (10.75 × 10^−6^ S/cm) increased several orders of magnitude up to 4.75 × 10^−4^ S/cm after deposition of C-Dots. These results are in accordance to what previously reported, in particular by H. Bi *et al*., about the potential of carbon dots on the enhancement of surface conductivity and many other properties^96–98^. Similar effects were also reported by us for the lead-free manganese ion modified ferroelectric ceramics K_0.5_Nb_0.5_NaO_3_^92^.

The illustration in Fig. 6b presents the model for the hybrid supercapacitor containing C-Dot_green_ decorated α-MnO_2_-PPy nanorods and rGO as a negative and positive electrode, respectively, separated with cellulose. C8H16BImI ionic liquid electrolyte was synthesized and characterizations were performed by ^1^H and 13C NMR (See supporting file and Figs S5, S6 and S7). The C-Dot_green_ decorated-MnO_2_-PPy nanorods and C8H16BImI ionic liquid electrolyte (Fig. S4) material have a crucial role in the capacitor device by enabling surface reactions and faster charge transfer^99, 100^. Alternative electrolyte would be LiPF_6_ but it cannot handle high voltage and material should not react with moisture. The hybrid supercapacitors presented here use the advantages of both capacitive behavior and the faradaic reaction to enhance energy density and power density instantaneously. Large surface area, high porosity, high electronic transport efficiency and structural stability are the benefits of C-related components^101, 102^. Here to describe the hybrid supercapacitor the Nyquist plot (Fig. 6d) were fitted to a modified Randles electrical circuit (Fig. 6c) by using the following elements: active Ohmic electrolyte resistance (*R*_1_), impedance of a faradaic reaction consists of an active charge transfer resistance (*R*_2_) and the double layer capacity (*C*). A constant phase element (CPE) has been used during the fitting procedure to correct the ideal capacitor behavior to a real one. Note that one can present the CPE of 45° as the Warburg diffusion element. The impedance spectra in Fig. 6d revealed a semicircle in the high-frequency region and a near-linear line in the low-frequency region. The semicircle can be related to the charge-transfer resistance *R*_2_ while the linear region is caused by Warburg diffusion element which is in this work described as CPE. At high frequencies, the intercept with the real axis (*Z*′) can treated as the electrolyte resistance *R*_1_. The Bode-plot in Fig. 6e shows the variation of the impedance as a function of the frequency (0.1 Hz–100 kHz) for hybrid supercapacitor. It confirms the validation of capacitance measurements while the curve follows the expected Kramers-Kronig relation which is an obvious increasing of the impedance at low frequency. Bode-plot of Randles circuit has three specific regions in Fig. 6e: (i). Above 1.5 kHz, magnitude approaches 5.4 Ω. The *R*_1_ dominates the region^103, 104^. (ii) Between 1.5 kHz and 20 Hz, capacitance (*C*) controls the impedance. Moreover, the maximum point of Z-phase between hybrid capacitor mechanisms is shown in Fig. 6e for 45 ° at 0.14 kHz which corresponds to the almost linear behavior (on the Bode plot of frequency versus impedance) with a slope of ~1. Finally, (iii) below 20 Hz the impedance begins a transition back towards resistive behavior as *R*_2_ becomes dominant. This transition is not complete even at down to 0.1 Hz.

As depicted in Table 3, the high area capacitance value can be read as 17.3 μF cm^−2^ at a working voltage of 3 V. Such a high value is completely related due to hybrid capacitance from the surface interaction between carbon related defects and C-Dots and/or rGO and of course related to the enhanced ion mobility of C8H16BImI electrolyte. Such intrinsic defects can act as reactive sites providing an effective charge transfer and good electrode-electrolyte interfaces that minimize the internal resistance of supercapacitor devices^105^. Here, we present geometric area due to the simplicity and accuracy reasons. Capacitance values reported in the literature for surface decorated MnO_2_ and carbon based materials including graphene and C-Dots vary between 0.08 and 50 µF/cm^2^, which is within the range that was obtained in this study^97, 106–108^. Note that, gravimetric specific capacitance of the MnO_2_/PPy/C-Dots will be investigated in future works as well. Thus, we omit such measurements while multiple additional effects influenced the capacity of supercapacitors such as redox, adsorption and intercalation which are not comparable via relative surface measurements.

**Table 3:** The parameters obtained from EIS data fitting for hybrid supercapacitor.

*R*_1_ [Ω]	*R*_2_ [Ω]	*C* [μFcm^−2^]	CPE [Ω^−1^s]	Working voltage [V]
5.4	18.7	17.3	0.0089	3

## Experimental Section

### Materials

Nile Red, Manganese sulfate monohydrate (MnSO_4_.H_2_O), potassium permanganate (KMnO_4_) and pyrrole were purchased from Sigma-Aldrich. Ethanol (96% pure. grad.) and other solvents were obtained from Fluka. All the reagents were analytical grade and used without further purification. Cola was purchased from a local supermarket. Octyl-bis(3-methylimidazolium) diiodide (C8H16BImI) was prepared according to previously reported procedures^109, 110^, and its molecular structure and NMR spectra were shown in Figs S5 and S6. Water used during the synthesis was purified using a Millipore Milli-Q system (18.2 MQ-cm).

### C-Dot Synthesis

Fluorescent Carbon Dots (C-Dots) synthesis was accomplished according to the method described previously^62^. 1 g. of C source mixture as described below was diluted in 1:10 ratio with MilliQ-water (Millipore Inc., Ω = 18 MΩ·cm) and dispersed in 2 mL of 1:1 water/ethanol in a Teflon oven vessel (approximately 20 mL inner volume). A homogeneous suspension was obtained without aggregation by vigorous mixing. The mixture was maintained at 300 °C for 30 minutes until a dark, caramelized, semi-solid texture was obtained. The resulting material was dissolved in 2 mL of MilliQ-water. The suspension was centrifuged at 6.000 rpm for 10 minutes, and the supernatant was passed through a 0.45 µm Millipore HV hydrophilic membrane filter for eliminating any impurities. The final product was vacuum-dried at 90 °C for overnight. Sample codes were shown in Table 1. Green emitting C-Dots (C-Dot_green_) were prepared directly using degassed Cola drink as carbon source without the utilization of any passivating agent. The synthesis of Blue emitting C-Dots (C-Dot_blue_) was achieved by mixing an equal volume of Nile Red (5 mg/mL) with Cola drink at the beginning. Red emitting C-Dots (C-Dot_red_) were synthesized following a hydrothermal synthesis route for preparation of reduced graphene oxide/C-dot_green_ hydrogels^65^. As prepared C-Dots_green_ (0.1 g) were dissolved in pre-synthesized graphene oxide (GOx) (2 mg/mL). The mixture was transferred into a Teflon-lined stainless steel autoclave, and heated at 180 °C for 12 h. Resulting material was immersed in hydrazine monohydrate (50%) in a a glass tube and were left for 8 h at 95 °C. Then, the resulting graphene oxide/C-dot_green_ hydrogels were washed via ultrapure water-cycles to remove the residuals. C-Dots were further collected from the washing water by centrifugation at 5000 rpm for 10 min and dried as mentioned above.

### Characterization of Carbon Dots

Ultraviolet-visible (UV-Vis) spectra of C-Dots were recorded using a UV-VIS spectrophotometer (Shimadzu UV-1800). Fluorescence properties were measured using a Varian Cary Eclipse Fluorescence Spectrophotometer. Quantum yields (QY) of each sample was calculated by taking quinine sulfate (QS) in 0.1 M H_2_SO_4_ as reference fluorophore. Quinine sulfate (QS) has the quantum yield of 0.54 (Ex. 360 nm). QY of each C-Dot is calculated following the procedure reported previously^111^. X-ray diffraction (XRD) analyses were performed with X-ray diffractometer (XRD) (Rigaku Smartlab Intelligent Americas, Texas USA). The XRD was recorded at the voltage of 40 kV with 2θ values between 8° and 80° at a scan rate of 2°/min. The dried powders of Carbon Dots were diluted with Mili-Q water to perform Fourier transform infrared (FTIR) spectroscopy (Perkin Elmer Frontier Waltham, A 02451 USA) and spectrum GX spectrometry within the wavenumber range of 400–4.000 cm^−1^. High-Resolution Transmission Electron microscopy imaging was performed using High-Resolution Transmission Electron Microscope (HRTEM) 200 kV with Field Emission (TECNAI G2 20 S-TWIN, FEI) to determine the size and morphology of the dispersed C-Dots and size analysis of the sample were carried out using *Image J*. Fluorescence spectra of particle solutions were measured using a Fluorescence Spectrophotometer (Varian Cary Eclipse.) Solid-state Raman spectra were recorded at room temperature (WITec GmbH Alpha300 R Raman Module). 785 nm wavelength laser was used under 50x magnification and 2.03 s integration times. X-band (9.86 GHz) EPR measurements were performed by BRUKER EMX spectrometer with the aid of a rectangular TE102 resonator from Bruker. The offset in the magnetic field and the exact g-factors in X-band measurements were determined with a polycrystalline DPPH (2-diphenyl-1-picrylhydrazyl) reference sample of well-known g-factor (g = 2.0036). The EPR spectral analysis has been performed using the WINEPR program from Bruker. For cooling (to liquid helium temperatures) an Oxford CF-935 cryostat was used. The temperature was regulated by a temperature controller (Oxford ITC-503). The following EPR experimental parameters were used: microwave power: 1 mW; modulation amplitude: 0.5 G; time constant: 163.84 ms; receiver gain: 2 × 10^4^.

### Hydrothermal synthesis of α-MnO_2_ Nanorods

α-MnO_2_ was synthesized by a hydrothermal method using MnSO_4_.H_2_O (0.3 g) and KMnO_4_ (0.6 g) mixture in 20 ml of ultrapure water. The homogeneous mixture was transferred into a Teflon-lined stainless steel autoclave and heated at 140 °C for 12 h. The autoclave was allowed to cool to room temperature, and then the precipitate was collected and washed four times with distilled water to remove any possible residual reactant. Finally, the α-MnO_2_ powder was dried in air at 40 °C^24, 112^.

### α-MnO_2_/C-Dot_green_ hybrid Nanorod Synthesis

Synthesis procedure starts with the preparation of α-MnO_2_ hybrid nanorods as explained in the previous section. Once nanorods synthesized, 60 mg of α-MnO_2_ and 60 mg of C-Dot_green_ were dispersed in 20 mL of ultrapure water; then the mixture was transferred into a Teflon-lined stainless steel autoclave, and heated at 140 °C for 12 h. The autoclave was allowed to cool to room temperature. The obtained product in hydrogel form was washed with distilled water to remove any possible residual reactant and then dried in a freeze dryer.

### α-MnO_2_/PPy Nanorod Synthesis

60 mg of α-MnO_2_ was dispersed in 20 mL of ultrapure water and sonicated. During sonication process, 200 µl of pyrrole added dropwise to this mixture. Then, the mixture was transferred into a Teflon-lined stainless steel autoclave, and heated at 140 °C for 12 h. The autoclave was allowed to cool to room temperature. The obtained product in hydrogel form was washed with distilled water to remove any possible residual reactant and then dried in a freeze dryer.

### *In situ* preparation of α-MnO_2_/C-Dot_green_ hybrid Nanorods

α-MnO_2_/C-Dot_green_ hybrid nanorods were synthesized by mixing reactants (0.3 g of MnSO_4_.H_2_O, 0.6 g of KMnO_4_) with 0.1 g of C-Dot_green_ in 20 mL ultrapure water. This mixture was sonicated and transferred into a Teflon-lined stainless steel autoclave, and heated at 140 °C for 12 h. After hydrothermal reaction, the product was collected, washed, and dried in a manner similar to that of α-MnO_2_.

### Electrochemical impedance spectroscopy (EIS) measurements

EIS measurements were carried out using a Versastat IV potentiostat (Princeton Applied Research) in atmospheric pressure at room temperature in the frequency range between 0.1 Hz and 100 kHz. For analyzing the spectra we used the Versa Studio V2.4 software. The amplitude of voltage modulation was set to 500 mV. In present case a four-point electrode-setup was used, so that the potentials for any electrochemical reactions that are occurring at the working electrode(s) are not being measured. The setup consisted of a copper foil for anode (thickness = 2 mm) and an aluminium foil for cathode (thickness = 0.5 mm) current collector on which the electrode material was coated. Both electrodes were separated by a cellulosic paper membrane. The setup was hold between two glass object plates, for a better adhesiveness

## Conclusions

In this study, multicolored (red, blue and green) fluorescent C-Dots were prepared through thermal synthesis using degassed Cola beverage as a C source; and optical and physicochemical properties were analyzed using various analysis methods. The synthesized α-MnO_2_/PPy hybrid nanorod was decorated with C-Dots as used for anode material for high performance hybrid SC. In addition to present work, other C rich materials can be used as C source such as waste water or exhaust filters of combustion vehicles or factory chimney filters. Liquid ionic membrane electrolyte octyl-bis(3-methylimidazolium)diiodide (C8H16BImI) were synthesized by N-alkylation reaction in order to enhance the electron transfer exceptionally in hybrid SC. C8H16BImI electrolyte is highly promising material for larger voltage window in the use of next-generation SC. Accordingly, conductivity and the ion-donating ability of C-Dots together with the capacitative property of α-MnO_2_ nanorods and C8H16BImI demonstrated markedly superior capacitive and rate performance compared with those standard electrode-electrolyte combinations. EPR and Raman spectroscopy results revealed the importance function of the defect centers in the performance of the SC device. Nowadays, SC are suffering plenty of limitation factors: High leakage current, thermal aging, high equivalent series resistance (ESR), low voltage window etc. To overcome such drawback one should consider the variety of materials in a smart way. Of course there are materials giving higher capacity values such as graphite however the performance of them in SC is limited and saturated. We believe new intelligent materials with alternative sources may have high impact in engineering to produce next generation SC while there is plenty of space on the materials selection compared to Li-ion batteries. Thus, such hybrid SC can be used as a standard high power source in electrical vehicles in the near future.

## Electronic supplementary material

Supplementary Info
