# Supplementary material for: High-Capacitance Hybrid Supercapacitor Based on Multi-Colored Fluorescent Carbon-Dots
Source: Sci Rep. 2017 Sep 11;7:11222. doi: 10.1038/s41598-017-11347-1 (PMC5593850; doi:10.1038/s41598-017-11347-1)
Supplement: Supplementary file 1 — Supplementary Info [file 41598_2017_11347_MOESM1_ESM.pdf]

Supplementary Materials

High-Capacitance Hybrid Supercapacitor Based on Multi-Colored Fluorescent Carbon-Dots

Rukan Genc[1,2]*, Melis Ozge Alas[1], Ersan Harputlu[2], Sergej Repp[3], Nora Kremer[3], Mike Castellano[3], Suleyman Gokhan Colak[2], Kasim Ocakoglu[2,4]*, Emre Erdem[3]*

[1] Department of Chemical Engineering, Engineering Faculty of Mersin University, Mersin University, TR-33343, Mersin, Turkey.

[2]Advanced Technology, Research, and Application Center, Mersin University, TR-33343, Mersin, Turkey.

[3]Institut für Physikalische Chemie, Albert-Ludwigs-Universität Freiburg, Albertstr. 21, 79104 Freiburg, Germany.

[4]Department of Energy Systems Engineering, Faculty of Technology, Mersin University, TR-33480 Tarsus, Mersin, Turkey.

*To whom correspondence should be addressed: Genc, R., E-mail: rgenc@mersin.edu.tr; Ocakoglu, K., E-mail: kasim.ocakoglu@mersin.edu.tr and Erdem, E., Email:emre.erdem@physchem.uni-freiburg.de

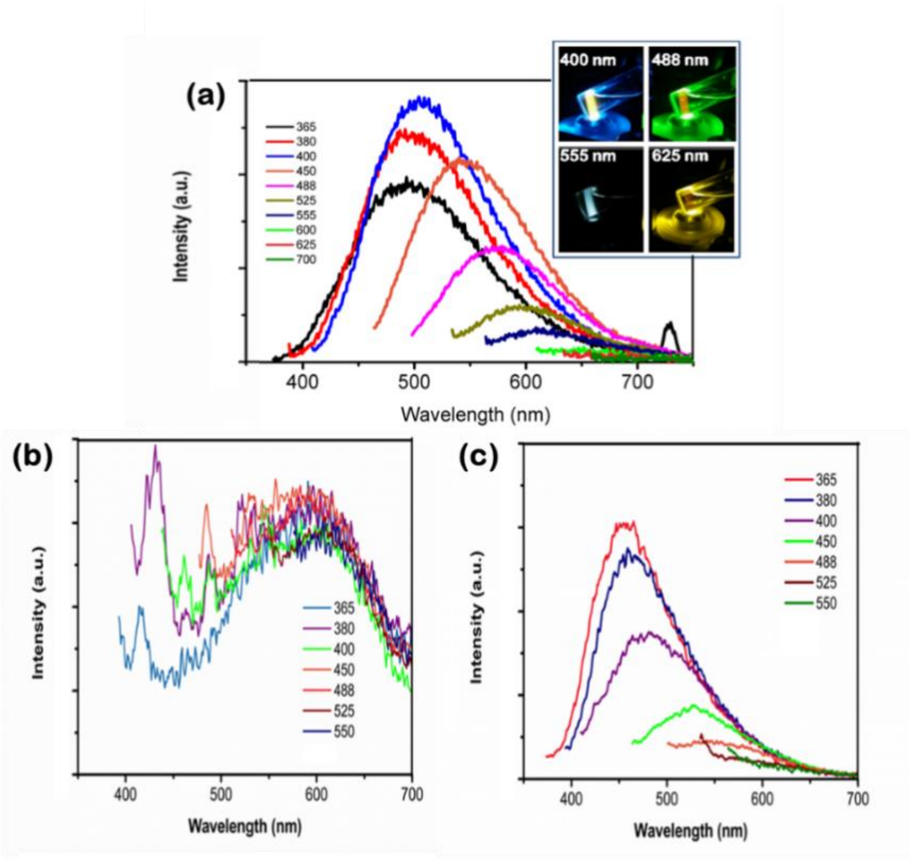

Figure S1. Excitation wavelength dependencies of C-Dots irradiated in the range of 400 nm and 750 nm: a) C-Dot$_{green}$, b) C-Dot$_{red}$ and c) C-Dot$_{blue}$. Inset figure is the fluorescence image of the nanoparticle solution irradiated with lasers having different excitation wavelengths.

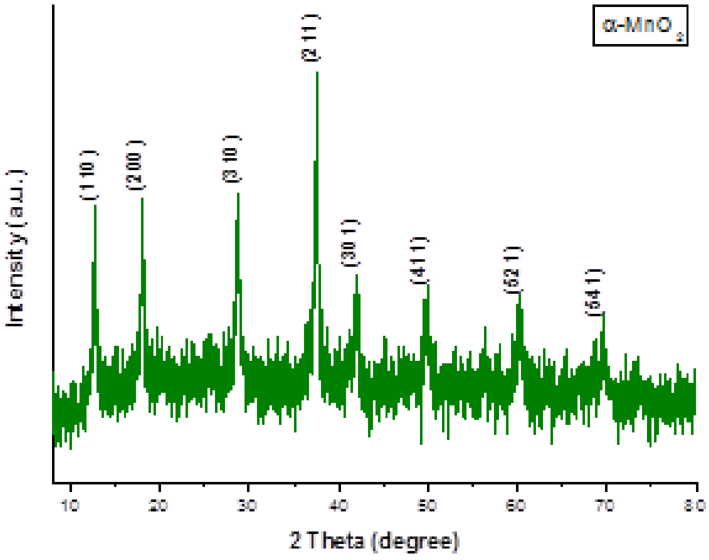

Figure S2. X-ray diffraction pattern of as-synthesized α-MnO_2 Nanorods.

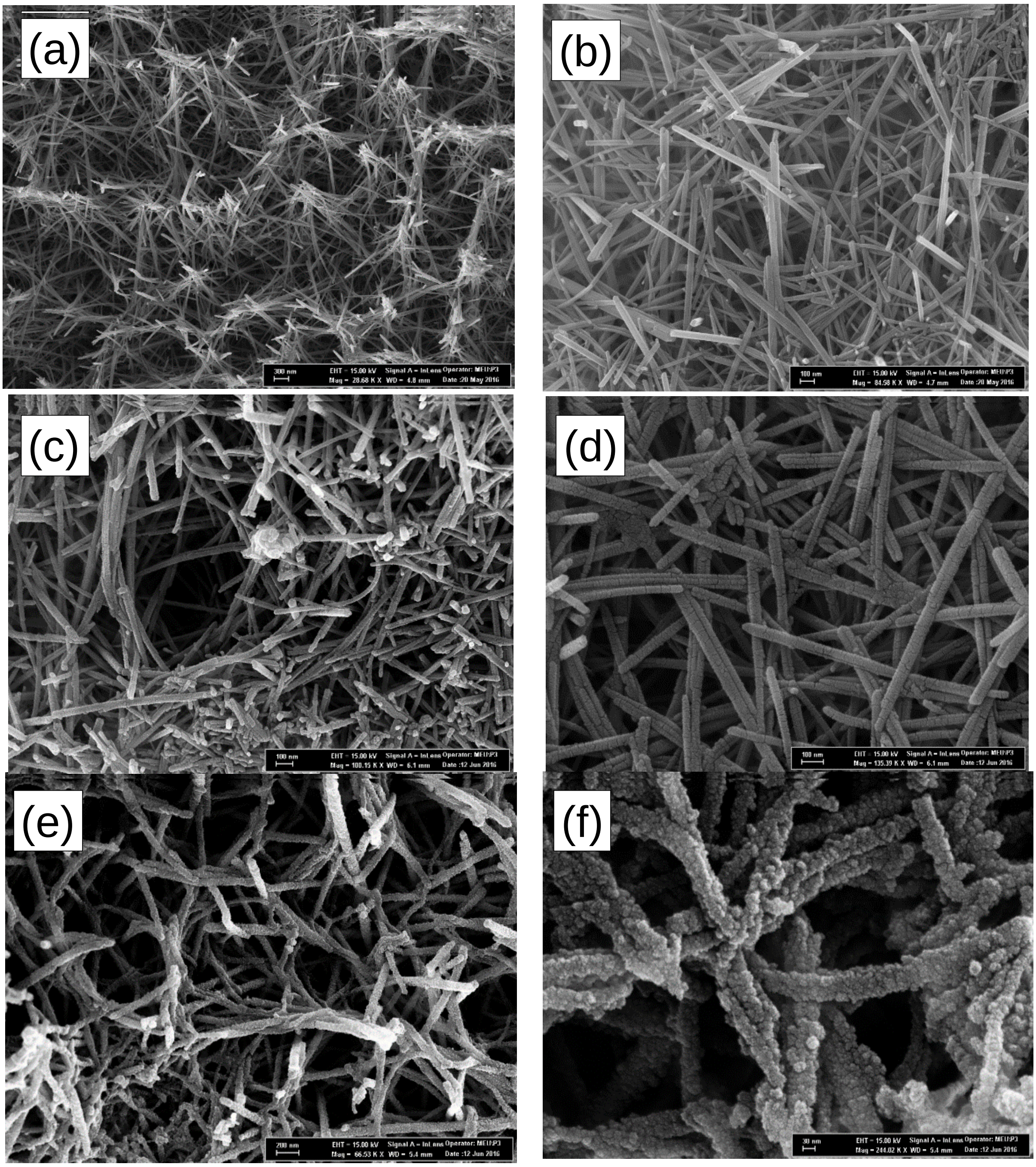

Figure S3. SEM images of one-dimensional α-MnO_2 nanorods and C-Dot_{green}/ α-MnO_2 nanohybrids synthesized through hydrothermal method: (a, b) as-synthesized α-MnO_2, (c, d) C-Dot_{green}/ α-MnO_2 hybrids nanorods, (e, f) α-MnO_2/polypyrrole/ C-Dot_{green} hybrid nanorods

FT-IR Analysis of C-Dots

C-Dot_{red} exhibited a sharper but weak peak at 3183 cm^{-1} associated with the amine groups. Bands in the region of 2750-3000 cm^{-1} are attributed to alkyl groups. The intensity of the transmittance at 2978 cm^{-1} (asymmetric stretching bands of methyl functionalities (-CH_3) of C-Dot_{red} increased in respect to the *sp*^{3} C-H stretching vibration peak centered at around 2882 cm^{-1}. This means that C-Dot_{red} has a methyl group dominated surface.[1] C-Dot_{blue}, on the other hand, showed a single peak centered at 2916 cm^{-1} assigned to the C-H stretching modes of hydrogenated carbon surface. Amide II band was observed in C-Dot_{green} at 1654 cm^1. C-Dot_{blue} also exhibited two strong peaks at 1379 cm^{-1} and 1586 cm^{-1} ascribed to C– H vibrations and C=C double bond stretching, respectively. These bonds appeared in moderate intensity at C-Dot_{red} while C=C bond almost disappeared in C-Dot_{green}. C-Dot_{red} exhibited several C–O stretching bands appeared in the 1000-1400 cm^{-1} range which implies the presence of high amount of residual hydroxyl groups and different modes of -C–O–C- (1140-1180cm^{-1}). The strong peak centered at 1010 cm^{-1} in C-Dot_{green} is assigned to the stretching vibration of C–OH of alcoholic groups and carboxylic acids.[2,3]

Synthesis of Octyl-bis(3-methylimidazolium)diiodide

Octyl-bis(3-methylimidazolium)diiodide was prepared according to the previously reported procedures (Figure S4).[4,5] A mixture of 1,8-diiodooctane (5.52 g, 15.1 mmol) and 1-methylimidazole (2.42 ml, 30.2 mmol) in 10 ml of toluene were heated to 110 ^{o}C for 12 h. The raw product was dissolved in CH_2Cl_2 and filtered. The yellowish viscous liquid was obtained by evaporating the filtrate to dryness in a rotary evaporator. ^{1}H and 13C NMR spectra were measured on a Bruker 400 MHz spectrometer. ^{1}H NMR (CH_3OD) ppm: 8.70 (s, 2H), 7.46 (s, 2H), 7.41 (s, 2H), 4.17 (t, J = 7.2 Hz, 4H), 3.87 (s, 6H), 1.84 (q, J = 7.2 Hz, 4H), 1.29 (s, 8H) (Figure S5). ^{13}C NMR (CH_3OD) ppm: 135.6, 123.60, 122.3, 49.6, 36.0, 29.2, 27.9, 25.3 (Figure S6).

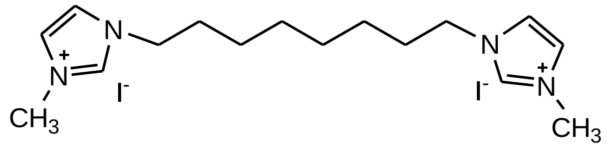

Figure S4. Molecular structure of octyl-bis(3-methylimidazolium)diiodide.

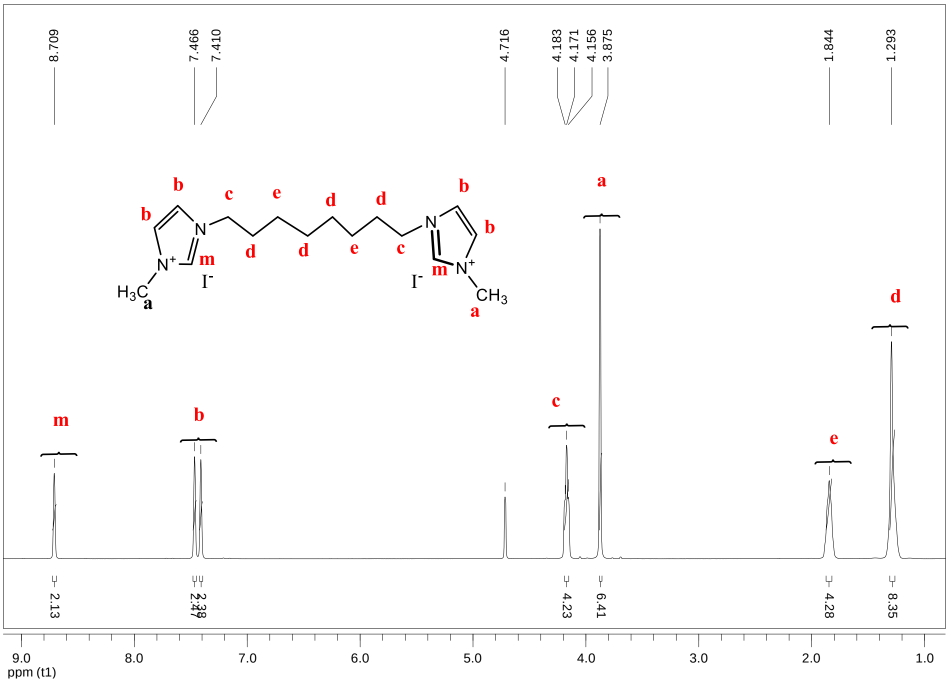

Figure S5. ^{1}H-NMR spectrum of octyl-bis(3-methylimidazolium)diiodide.

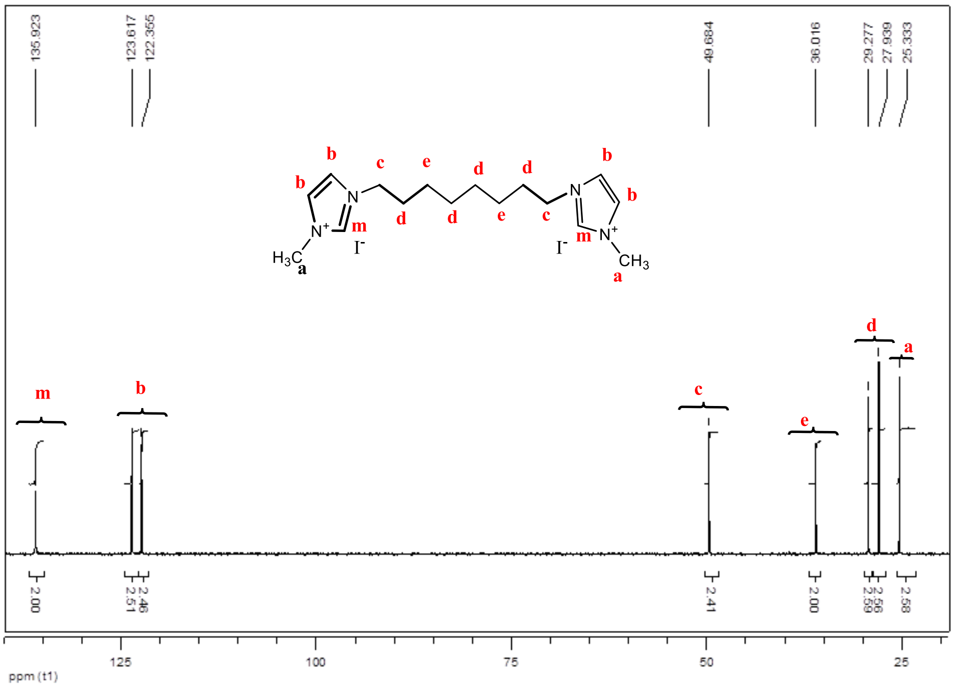

Figure S6. ^{13}C-NMR spectrum of octyl-bis(3-methylimidazolium)diiodide.

Synthesis of reduced graphene oxide (rGO)

The graphene oxide was synthesized using "modified Hummer's method". [6] To do so, natural graphite flakes (0.618 g, 1 wt%), $NaNO_3$ (0.543 g), H_2SO_4 (97-99%, 23 mL) and $KMnO_4$ (2.985 g, 5 wt%) were mixed in an ice bath under strong magnetic stirring. At the end of the exothermic oxidation, the black-green reaction mixture was stirred at room temperature (RT) for 51.5 h. During the stirring, an increase of viscosity as well as a slow colour change from black-green to brown-orange has been observed. The reaction mixture has been diluted with deionized water (46 mL) and heated at 90 °C for 1.5 h. After cooling down to RT, H_2O_2 (30%, 10 mL) was added to the light brown-orange reaction mixture to remove unreacted $KMnO_4$. The oxidized graphite was washed with HCl (1M, 25 mL) followed by centrifugation (2500 rpm, 10 min). For further purification, the light brown solid was washed with a H_2O/EtOH-mixture (1:5, 18 mL) and centrifuged (4000 rpm, 15 min) again. The synthesized solid was dispersed in deionized water (160 mL), sonicated for 2 h and centrifuged (4000 rpm, 10 min). Finally, to improve the exfoliation of the black graphene oxide, the sample was sonicated for another 2 h. Resulting rGo was monitored with TEM (Figure S7).

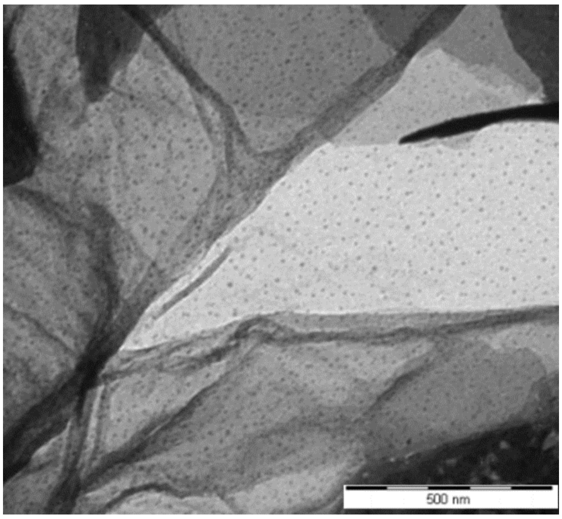

Figure S7: TEM photograph of synthesized rGO. It is clearly seen that several rGO sheets formed.

Temperature dependent EPR spectra of the C-Dot$_{red}$

We investigated the temperature dependent EPR spectra of the C-Dot$_{red}$ sample in order to gain more information about the behavior of red C-Dots. The temperature dependent EPR of blue and red C-Dots revealed non-Curie behavior so that we do not present here. This is also one indication of high number of surface defect in this samples while surface defects do not carry the character of the crystal so that they do not have typical Curie paramgnetism.[85] Thus we only present temperature dependent X-band EPR measurements from 250 K down to 10 K for C-Dot$_{red}$. The temperature evolution of the EPR spectra of a C-Dot$_{red}$ sample is presented in the inset of Fig. 5c. The EPR signal of C-Dot$_{red}$ exhibits the expected temperature dependence given by Curie's law. Curie-type behavior suggests that the number of defects that exist initially located on the core of the sample. We suppose that C-Dot$_{red}$ might be good radical trapping center, keeping spins more delocalized.

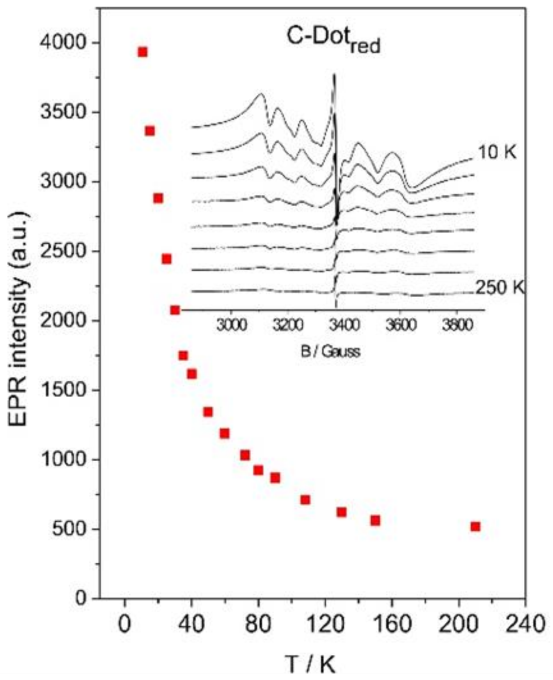

Fig. S8. The plot of X-band EPR inverse intensity with respect to temperature for C-Dot$_{red}$ (*inset*: temperature dependent EPR spectra measured between 10-250 K).

Table S1. EDX Analysis of C-Dot$_{Green}$

Element	Concen tration	Intensity correction	Mass %	Mass % sigma	Atom %
C K	0.84	1.1639	0.72	0.01	56.94
O K	0.43	0.5960	0.72	0.01	42.76
Na K	0.00	0.7571	0.00	0.00	0.10
P K	0.01	1.3008	0.01	0.00	0.17
S K	0.00	0.9489	0.00	0.00	0.03
Sum.			1.46		

Table S2. EDX Analysis of C-Dot$_{Blue}$

Element	Concen tration	Intensity correction	Mass %	Mass % sigma	Atom %
C K	0.49	0.8216	0.59	0.02	56.92
O K	0.28	0.5129	0.54	0.01	39.18
Na K	0.01	0.7819	0.01	0.00	0.47
P K	0.01	1.3249	0.01	0.00	0.19
Cl K	0.03	0.8382	0.04	0.00	1.17
K K	0.07	1.0387	0.07	0.00	2.06
Sum			1.26		

Table S3. EDX Analysis of C-Dot$_{Red}$

Element	Concen tration	Intensity correction	Mass %	Mass % sigma	Atom %
C K	0.38	0.5781	0.64	0.01	51.18
O K	0.30	0.4591	0.67	0.00	40.37
Na K	0.01	0.7697	0.01	0.00	0.43
P K	0.02	1.3448	0.01	0.00	0.39
S K	0.01	0.9858	0.01	0.00	0.19
Cl K	0.08	0.8516	0.09	0.00	2.53
K K	0.21	1.0363	0.20	0.00	4.91
Sum			1.63		

References

1. Sha, Y. *et al. Hydrothermal synthesis of nitrogen-containing carbon nanodots as the high-efficient sensor for copper(II) ions*. *Materials Research Bulletin* **48,** (2013).

2. Tripathi, K. M., Tyagi, A., Ashfaq, M. & Gupta, R. K. Temperature dependent, shape variant synthesis of photoluminescent and biocompatible carbon nanostructures from almond husk for applications in dye removal. *RSC Adv.* **6,** 29545–29553 (2016).

3. Baker, S. N. & Baker, G. A. Luminescent Carbon Nanodots: Emergent Nanolights.

4. Erten-Ela, S. & Ocakoglu, K. Iridium dimer complex for dye sensitized solar cells

using electrolyte combinations with different ionic liquids. *Mater. Sci. Semicond. Process.* **27,** 532–540 (2014).

5. Zafer, C., Ocakoglu, K., Ozsoy, C. & Icli, S. Dicationic bis-imidazolium molten salts for efficient dye sensitized solar cells: Synthesis and photovoltaic properties. *Electrochim. Acta* **54,** 5709–5714 (2009).

6. Lei, Z., Zhang, J., Zhang, L. L., Kumar, N. A. & Zhao, X. S. Functionalization of chemically derived graphene for improving its electrocapacitive energy storage properties. *Energy Environ. Sci.* **9,** 1891–1930 (2016).
